# Host Transcriptomics Reveal Reduction in Defence‐Reproduction Trade‐Offs During Coinfection

**DOI:** 10.1111/mec.70124

**Published:** 2025-10-13

**Authors:** Ian Will, Emily J. Stevens, Kayla C. King, Kieran A. Bates

**Affiliations:** ^1^ Department of Biology University of Oxford Oxford UK; ^2^ Department of Zoology University of British Columbia Vancouver British Columbia Canada; ^3^ School of Life Sciences Keele University Newcastle‐under‐Lyme UK; ^4^ Department of Microbiology & Immunology University of British Columbia Vancouver British Columbia Canada; ^5^ Blizard Institute, Faculty of Medicine and Dentistry Queen Mary University of London London UK

**Keywords:** *Caenorhabditis*, defence, disease, *Leucobacter*, parasites, trade‐offs

## Abstract

During infection, hosts may shift resources away from reproduction towards immune defence. It is unclear to what degree these costly trade‐offs can be alleviated during protective coinfections, whereby antagonism between parasites reduces disease severity. We used transcriptomics to assess the extent to which host gene expression reflected the effect of protection and whether reducing or increasing investment in immunity carried costs to reproduction. Virulent infections by *Leucobacter musarum* bacteria elicited greater trade‐offs in nematode hosts compared to the naturally coinfecting ‘protective parasite’ *Leucobacter celer* . We further found that coinfection attenuated host investment in pro‐immune trade‐offs, without significantly changing which host genes were involved. We then sought to understand if this attenuated host response would be consistent with possible mechanisms of inter‐parasite competition. Our chromosome length genome assemblies for both parasite species revealed that protective coinfection may operate by competition for public goods, such as siderophore‐mediated uptake of metal ions (e.g., iron) or colonisation of the host cuticle. Ultimately, we show that competition between coinfecting parasites can complement endogenous host defences and ease the reproductive costs of fighting harmful infection.

## 1 Introduction

Infection drives myriad host defence strategies to minimise costs to health and fitness. Hosts may avoid, resist or tolerate parasites (Baucom and De Roode 2011; Medzhitov et al. 2012) using immunological, behavioural and symbiont‐mediated defences (Clay 2014; Lei et al. 2024; Nürnberger et al. 2004). However, defence comes at a cost. Across taxa there is evidence for tension in allocating limited resources to the demands of defence, reproduction and body condition (Anderson and Pukkila‐Worley 2020; Moret and Schmid‐Hempel 2000; Obeso 2002; Reznick 1982; Stearns 1989; Vorburger and Perlman 2018). For example, mating effort can lead to reduced immune function and lifespan in insects (Fedorka et al. 2004), and in plants, the production of defensive compounds can come at a cost to growth (Züst et al. 2015). Defence‐reproduction trade‐offs have further been supported by experimental studies demonstrating that removal of invertebrate host germlines enhances immunity (Alper et al. 2009; Miyata et al. 2008; Rae et al. 2012; Rodrigues et al. 2021; Tekippe and Aballay 2010).

These fitness trade‐offs may be reduced when the virulence, or disease severity, of a parasite infection is lessened. Virtually all organisms are colonised by multiple organisms, spanning the mutualist‐parasite continuum (Hoarau et al. 2020). Coinfecting symbionts within a host can compete with each other in three major modes: exploitative competition (e.g., racing to consume resources), interference competition (e.g., molecular warfare) or apparent competition (e.g., modification or induction of host immunity) (Alizon et al. 2013; Ashby and King 2017; Ramesh and Hall 2025; Rolhion and Chassaing 2016). The result of competitive coinfections can lead to divergent outcomes at ecological and evolutionary timescales, including ‘protective coinfections’ whereby virulence is reduced (Ashby and King 2017; Clay 2014; Clay and Rudolf 2019; Rolhion and Chassaing 2016). For example, protective coinfections have been shown between microbes competing for public goods such as metairon in the host environment (i.e., exploitative competition) (Ford et al. 2016), by production of antimicrobial superoxides (i.e., interference competition) (King et al. 2016), and by immune priming their hosts (i.e., apparent competition) (Hoang et al. 2024). Protection from microbial symbionts can cause plastic host divestment of specific defences against parasites that drive virulent infections (Drew and King 2022; Ford and King 2021), and ultimately reduce selection on defence mechanisms (Ashby and King 2017; Bates et al. 2021; King and Bonsall 2017; Martinez et al. 2016). The impact of these protective coinfections on the fitness trade‐offs hosts make to survive and/or fight infection is unclear.

To investigate such trade‐offs, we measured host transcriptional responses in an animal‐microbial parasite system that leverages naturally coinfecting bacteria in *Caenorhabditis* nematodes (Clark and Hodgkin 2015). Compared to infection by *Leucobacter musarum* (CBX152), coinfection with *Leucobacter celer* (CBX151) attenuates the overall virulence in the model host organism *Caenorhabditis elegans* (N2 line) (Bates et al. 2021; Hodgkin et al. 2013). Context plays a key role in delimiting when we consider these symbionts as ‘net’ parasites or net protectors (Drew et al. 2021). Although *L. celer* infection is highly virulent with certain environmental or host conditions (Hodgkin et al. 2013), in the present experimental design, this bacterium confers net benefits to host survival during coinfection with the more harmful *L. musarum*. We measured host gene transcription at early and late infection timepoints to profile stage‐specific responses across single and coinfection treatments. To better interpret host responses in terms of the mechanism of protection during coinfection, we assembled and annotated chromosome‐length genome assemblies of both *Leucobacter* species. We then inferred possible mechanisms of virulence and microbial competition.

We hypothesised that hosts protected by coinfection could (i) outsource immunity, (ii) combine typical endogenous defences with coinfection, or (iii) complement protection with a distinct or increased coinfected‐host response (Hrček et al. 2018; Parker et al. 2011). These host responses would predict either reduced, constant, or enhanced (increased or qualitatively different) immuno‐transcriptional profiles, respectively. In turn, host trade‐offs between defence and reproduction could become less or more severe (i.e., hypotheses i and iii). We also assessed whether the change in the host response was consistent with likely modes of parasite interactions and protective coinfection identified from our genomic analyses (Alizon et al. 2013; Ashby and King 2017). Testing these hypotheses produced insights into fundamental interactions of hosts with symbionts spanning the mutualist‐parasite continuum.

## 2 Methods

### 2.1 Host‐Microbe System and Infection Assays

We maintained *C. elegans* nematodes on Nematode Growth Medium (NGM) plates colonised by *Escherichia coli* (OP50) as a food source (Stiernagle 2006). Virulence was driven by *L. musarum*. Host protection was conferred by *L. celer* . Infections of *C. elegans* with *Leucobacter* generally followed the methods detailed in Bates et al. (2021). We age‐synchronised nematodes by isolating nematode eggs with a bleach wash and hatching larval stage 1 (L1) larvae in M9 buffer (Stiernagle 2006). These L1 nematodes then grew on *E. coli* plates until stage L4. We grew all three bacteria overnight from frozen glycerol stocks in shaking lysogeny broth (LB) (171 mM NaCl, 141 mM tryptone, and 5 g/L yeast extract) cultures at 30°C ( *E. coli* ) or 25°C (*Leucobacter*) to approximate optical densities (600 nm) of 1.0 for *E*. *coli* and 0.3 for *Leucobacter*. To create infection exposure plates, we grew bacterial lawns on 9 cm NGM plates inoculated with 80% *E. coli* culture and 20% *Leucobacter* culture, by volume (200 μL total). Four treatment types were made: control (80% *E. coli* , 20% phosphate buffered saline), ‘LC’ (80% *E. coli* , 20% *L. celer* ), ‘LM’ (80% *E. coli* , 20% *L. musarum*) and coinfection (80% *E. coli* , 10% *L. celer* , 10% *L. musarum*). All plates (biological replicates) were incubated for 24 h at 25°C. We then transferred 1500 L4 nematodes to each of eight replicate plates per treatment and incubated all plates at 25°C.

We collected experiment samples at two timepoints, corresponding to early infection (10 h) and late infection (20 h). We chose these times as appreciable *C. elegans* mortality has been observed with these *L. musarum* infection methods by 24 h (Bates et al. 2021). Per sampling time, we collected nematodes from four replicate plates per treatment by flooding plates with 5 mL of M9 buffer. Samples were washed four times with M9 buffer by centrifugation. We suspended washed nematodes in a final volume of 800 μL of ice‐cold RNA Shield (Zymo Research) and immediately homogenised them by bead beating at 2800 rpm (Disruptor Genie, Scientific Industries) for 5 cycles of 2 min disruption—2 min ice incubation. Samples were then frozen at −80°C until RNA extraction.

Total RNA was extracted from samples using the Quick‐RNA Minprep Plus Kit (Zymo Research) following the manufacturer's protocol. Total RNA was sent to the CGR for RNAseq library preparation and Illumina sequencing. At the CGR, rRNA was depleted using the RiboZero Plus kit (Illumina) to enrich mRNA. Paired‐end, indexed, strand‐specific RNA libraries were prepared using the Ultra II Directional kit (NEB). Libraries were sequenced on a NovaSeq (Illumina) to produce 2 × 150 bp reads. Approximately 50–70 million reads were produced per sample. The CGR performed initial read trimming with Cutadapt (v. 1.2.1) using a 3 bp matching threshold to begin trimming (Martin 2011). The CGR also quality trimmed reads using Sickle (v. 1.200) with a Q20 window and minimum read length of 15 bp (Joshi and Fass 2011).

### 2.2 RNAseq Analysis to Identify Genes of Interest

We assigned reads (paired only) to *C. elegans* , *L. celer* , *L. musarum* or *E. coli* for quantification. Host RNA reference transcripts were available from NCBI under accession GCF 000002985.6. We generated transcript sequences for all three bacterial species using GffRead (v. 0.12.7) (Pertea and Pertea 2020) using the *Leucobacter* assemblies presented here and *E. coli* assembly GCF 009496595.1. Using Salmon (v 1.4.0), we pseudo‐mapped reads and quantified RNA transcript abundances using a concatenated all‐organisms transcriptome (Patro et al. 2017). From those mappings, we then separated transcript abundance data for the host and each of the three bacteria. Approximately 20–35 million read pairs mapped to the host transcriptome per sample, and we associated transcripts with gene names using tximport (v. 1.30.0) in RStudio (v. 2023.09.1) with R (v. 4.3.2) (R Core Team 2022; RStudio Team 2020; Soneson et al. 2016). For LC and coinfection treatments, we found 7000–350,000 *L. celer* reads per sample. For LM and coinfection treatments, we found 34,000–336,000 *L. musarum* reads per sample. Across all treatments, we found 3000–900,000 *E. coli* reads per sample. We considered the sequencing depth of both *Leucobacter* species too low for a robust analysis and therefore only mapped reads to bacterial transcriptomes to prevent non‐specific mapping to the host transcriptome. We discarded counts from non‐mRNA transcript sources such as residual rRNA or non‐coding piRNA.

Using DeSeq2 (v. 1.42.0), we normalised the count data and calculated pairwise differential gene expression between experimental treatments (Love et al. 2014). We selected up‐ and downregulated differentially expressed genes (DEGs) from Wald tests with a null hypothesis of < |1.5|‐fold change in gene expression between treatments, with a Benjamini–Hochberg adjusted false detection rate of *p* ≤ 0.05. The number of successfully tested *C. elegans* genes passing DeSeq2 independent filtering was in the range of 16,785–18,292, depending on the specific pairwise treatment comparison. We examined sample clustering by treatment with principal component analyses using DeSeq2 normalised and rlog transformed (option blind = False) gene counts in R and plotted with ggplot2 (v. 3.4.4) (Wickham 2016).

With WGCNA (v. 1.72–1), we created host gene co‐expression network modules correlated to experimental treatment types (Langfelder and Horvath 2008) (Supplemental Data S1). We reduced spurious testing by removing lowly expressed genes that had zero counts in over 28 (of 32) samples, leaving 18,782 analysed genes. We constructed a signed‐hybrid gene network using biweight midcorrelations (‘bicor’) of rlog transformed expression data, with recommended options for binary categorical trait data. We selected a soft power threshold of five where topology fit was > 0.9 and mean connectivity < 1000, in line with standard use of the WGCNA package (Langfelder and Horvath 2008). To identify modules of interest, we conservatively required that (i) the module eigengene had a significant (*p* ≤ 0.05) and strong (correlation ≥ |0.5|) association with LC, LM or coinfection treatment type and (ii) that module was not significantly correlated to contrast group samples (*p* ≤ 0.05) with the same sign (positive or negative correlation) as the infection treatments. The contrast group for LC or LM modules was the control treatment. For coinfection, the contrast was LM. We additionally calculated the amount of gene expression variance explained per module eigengene using the WGCNA package.

To leverage our RNAseq data for functional inference, we assumed gene transcription correlated to translation and protein function. Such a link between mRNA and protein has been shown for DEGs responding to experimental treatment (Koussounadis et al. 2015). Although, in some cases, gene transcription can poorly relate to translation due to post‐transcriptional/translation regulation (Vogel and Marcotte 2012). Our choice to sequence populations of nematodes (replicate plates) rather than individuals was based on practical constraints, favouring higher power detection of DEGs (Wang et al. 2022) and to best replicate the experimental design of the work (Bates et al. 2021) that inspired the present study.

### 2.3 GO Term Enrichment Analyses on Genes of Interest

We conducted multiple GO term enrichment analyses using either gene set enrichment analyses (GSEA) with gene expression rankings or hypergeometric enrichment analyses with gene classifications (Benjamini‐Hochberg multiple testing adjusted FDR *p* ≤ 0.05) (The Gene Ontology Consortium et al. 2023). The GSEAs used genes ranked by fold‐change expressions calculated by DEseq2. A benchmarking study has indicated that GSEA is a high‐sensitivity method suitable for exploratory analyses (Mathur et al. 2018), even though some caution against false‐positives has been suggested from other tests (Tarca et al. 2013). We used WGCNA module membership to classify genes of interest relative to the background transcriptome in hypergeometric enrichment analyses. For all enrichment analyses, we used clusterProfiler (v. 4.12.0) (Yu et al. 2012). Gene annotations for *C. elegans* were obtained from R package org.Ce.eg.db (v. 3.19.1) (Carlson 2019). We reported enriched biological process GO terms here and included additional results from the molecular function and cellular component ontologies in Data S2. We visualised enriched GO terms as networks of annotations connected by their relatedness. Using Revigo (v. 1.8.1), we reduced visual clutter by only displaying a representative term for redundant, closely related terms (0.7 similarity threshold) (Supek et al. 2011). GO term networks were produced by Revigo and we finalised them in Cytoscape (v. 3.10.2) (Shannon et al. 2003). Network layouts were prefuse force directed by edge weight and then manually adjusted for readability and to group isolated nodes. All figures were finalised with Inkscape (v. 1.3.2).

### 2.4 Bacterial Genome Assemblies

We produced *de novo* genome assemblies from HiFi PacBio sequences for both *L. celer* and *L*. *musarum* bacteria (Gram‐positive, Phylum Actinomycetota). For both species, single colonies were selected from LB agar lates and grown to a saturated culture for 3 days in 15 mL of LB at 25°C with gentle shaking. Cells were then collected by centrifugation, and DNA was extracted using the NEB Monarch Genomic DNA extraction kit (T3010) and protocol, with an additional overnight chemical lysis step using lysozyme and mutanolysin to digest cell walls. Sample quality was validated and sequenced by the Centre for Genomic Research (CGR) at the University of Liverpool. Both *Leucobacter* species samples were multiplexed on a Sequel II instrument to produce over 650,000 Q20+ reads with median length above 18 kbp for each of *L. celer* and *L. musarum*. We then randomly down‐sampled each set of sequencing data to an estimated 100× fold coverage of read data using seqtk (v. 1.3). Genome size estimates prior to assembly were based on published short‐read assemblies available for both *L. celer* (RefSeq GCF_001273835.1) and *L. musarum* (RefSeq GCF_001273845.1) (Clark and Hodgkin 2015), corroborated by k‐mer counting data analysis using jellyfish (v. 2.3.0) and GenomeScope (v. 1.0) on our read data.

To assemble reads, we used Flye (v. 2.9.3) with default settings and suppressed alternative contigs for these haploid bacterial assemblies. We performed two additional iterations of random down‐sampling and assembly per species and verified that the assemblies were consistent regardless of the random read subset used. We annotated and assessed the assemblies using BUSCO (v. 5.6.1) (reference database micrococcales_odb10), PGAP (v. 2023‐10‐03.build7061), eggNOG‐Mapper (v. 2.1.12), InterProScan (v. 5.66–98.0), geNomad (v. 1.8.0), PathoFact (v. 1.0, ORF branch), MMseqs2 (v. 13) to search for protein matches to the virulence factor database (VFDB, v. July 2024), SOCfinder (v. 1.2) and FeGenie (v. 1.2) (Table S1 and Data S3) (Belcher et al. 2023; Camargo et al. 2023; Cantalapiedra et al. 2021; de Nies et al. 2021; Garber et al. 2020; Jones et al. 2014; Li et al. 2021; Liu et al. 2022; Manni et al. 2021; Steinegger and Söding 2017). BUSCO uses ‘universal’ orthologs as a benchmark for genome assembly completeness (Manni et al. 2021). PGAP, eggNOG‐Mapper and InterProScan are well‐established methods to annotate gene models, descriptions, protein domains and gene ontology (GO) terms (Cantalapiedra et al. 2021; Jones et al. 2014; Li et al. 2021). Using a curated database of chromosomal, plasmid and viral marker genes, geNomad detects possible proviral and plasmid sequences, producing the summary confidence metrics ‘virus score’ and ‘plasmid score’ (scales 0–1) (Camargo et al. 2023). PathoFact leverages a custom database of virulence protein models (largely derived from the VFDB) and a random forest classifier to identify likely virulence factors such as adhesion, surface marker, secretion machinery and toxin proteins (de Nies et al. 2021). We considered PathoFact hits selected by both the protein model matching and random forest sub‐analyses to have ‘full’ support, while ‘partial’ hits were supported by only one. For our direct query of the VFDB with MMseqs2, we used the experimentally supported core virulence factor list (Liu et al. 2022). To annotate potential ‘social genes’ that could offer clues as to how these bacterial species interact, we annotated genes with SOCfinder. Following upon siderophore‐related annotations from SOCfinder, we further identified possible siderophore synthesis genes with FeGenie and associated FeGenie outputs to other gene annotations via MMseqs2. We only used FeGenie to annotate putative siderophore synthesis genes as SOCfinder did not detect any siderophore synthesis gene clusters.

## 3 Results

### 3.1 Host Responses to Coinfection Were Driven by the More Harmful Parasite

**FIGURE 1:**
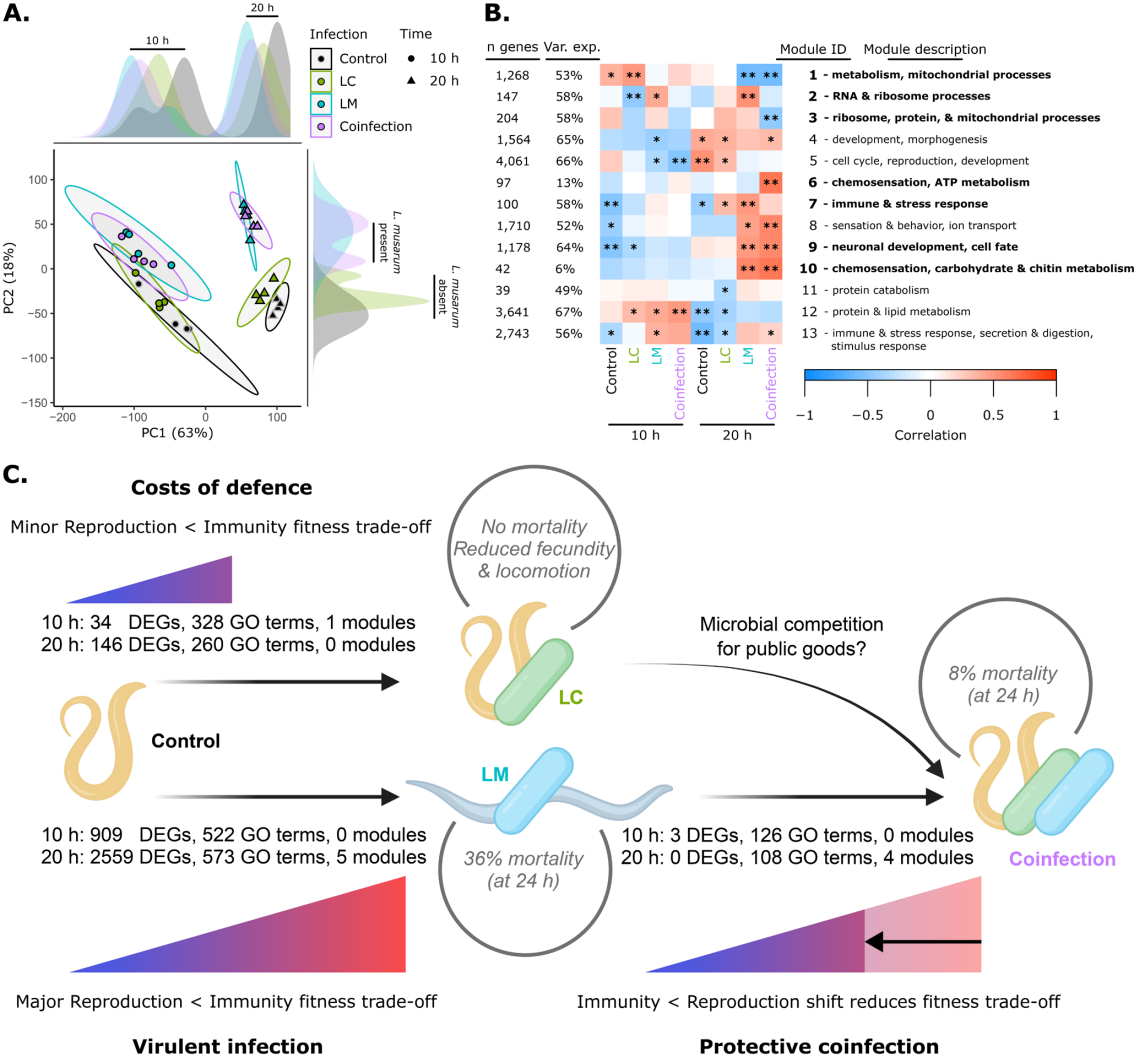
Transcriptome‐wide host gene expression differentiated infections. (A) The PCA of host transcriptomes showed that treatment timepoint and the presence of *L. musarum* drove host gene expression. Early (10 h) and late (20 h) timepoints clustered separately along PC1, which accounted for the most sample variation (63%). Principal component 2, largely tied to infection status, accounted for an additional 18% of sample variation. Gene expression profiles of hosts in control and LC treatments were highly similar, as were LM and coinfection samples (PC2). Density plots of PC1 (top) and PC2 (right) values are displayed along the axes to highlight these major groupings of samples by time and *L. musarum* presence. (B) A heatmap of WGCNA module eigengene value to sample type correlations highlights modules of relevance to infection. Significant module to treatment correlations (*p* ≤ 0.05) are marked as ‘*’ if the correlation was weak (< |0.5|). If the correlation is both strong (≥ |0.5|) and significant (*p* ≤ 0.05), we marked it as ‘**’. We focused on modules that were strongly correlated (**) to a single‐infection treatment without a significant correlation (* or **) of the same sign (positive or negative) to control samples at that time. We focused on modules with strong correlation to coinfection that contrasted with time‐matched LM treatments. This highlighted seven modules 1, 2, 3, 6, 7, 9 and 10 (shown in bold). Values for ‘Var. exp.’ give the percent of gene expression variance explained by the module eigengene. Network module descriptions are brief summaries of enriched biological process GO terms (full annotations for every module available in Data S2). (C) Hosts harbouring the protector only (LC) paid minor fitness costs compared to hosts with virulent single infections (LM). Coinfection attenuated the severity of fitness trade‐offs relative to LM infections alone, reducing expression of immune genes and increasing expression of reproduction and development genes. Notably, the differences between LM and coinfection treatment are primarily due to the degree of change at the gene group level (GSEA GO terms or WGCNA modules), rather than which DEGs respond to infection. Our bacterial genome assemblies suggested alternative mechanisms of infection virulence by the *Leucobacter* species and that protective coinfection may be mediated in part by microbial competition for public goods (i.e., iron or host cuticle space). Straight arrows indicate gene expression contrasts between treatment types with total counts of significantly different DEGs, GSEA GO terms and WGCNA modules given for both early (10 h) and late (20 h) timepoints. The trade‐off gradients indicate the severity of immunity‐reproduction trade‐offs inferred by gene expression (e.g., from control worms to LM treatment, showing moribund hosts as grey nematodes). Median mortality and morbidity data (grey circles) are from previous works (Bates et al. 2021; Hodgkin et al. 2013). Figure panel (C) was first drafted in BioRender (lab, K. (2025) BioRender.com/92xzjjz).

Multiple analyses revealed strong shifts in host gene expression based on sampling time and the presence of *L. musarum*, with fewer host changes caused by *L. celer* . A PCA of host gene expression data showed time (clustered by PC1, 63%) and infection (clustered by PC2, 18%) to be major drivers of treatment variation (Figure 1A). At both early (10 h) and late (20 h) timepoints, PC2 clustered treatments containing *L. musarum* (i.e., LM and coinfection) together, largely apart from control or LC (i.e., *L. celer* alone) treatments (Figure 1A). Broadly, this pattern indicated that the presence of *L. musarum* is the principal driver of the host response to infection compared to *L. celer* . We also used a co‐expressed gene network approach (WGCNA) to select gene modules strongly correlated with infection treatments. Ten of thirteen gene expression network modules distinguished infected hosts from controls (Figure 1B). All 10 clearly contrasted *L. musarum* (co‐)infected hosts from uninfected controls, with only one also distinguishing LC from controls. As a third method of characterising changes at the gene group level, we used GSEAs. These analyses associated functional GO term annotations with gene groups based on expression level rankings in pairwise comparisons of treatments. We again found LM and coinfection to elicit more change than LC treatments relative to control nematodes, with about 200 more enriched GO terms at either early or late infection times (Figure 1C and Table S2). At the individual gene level, the DEG analysis between early and late control nematodes showed large changes in host gene expression by time (2895 DEGs) that appeared to be almost entirely related to development (Data S2). We therefore only directly compared infection treatments within the same sampling timepoint to avoid conflating signals between developmental time and infection. DEG counts highlighted the greater impact of *L. musarum* infection over *L. celer* . Relative to controls, LM and coinfection treatments produced many DEGs at both early (909 or 886 DEGs, respectively) and late times (2559 or 2332 DEGs); while LC treatment was far less impactful (34 and 146 DEGs at early and late times, respectively) (Figure 1C and Table S3).

### 3.2 Virulent Single Infection Promoted Host Antimicrobial Immunity Over Reproductive Investment

**FIGURE 2:**
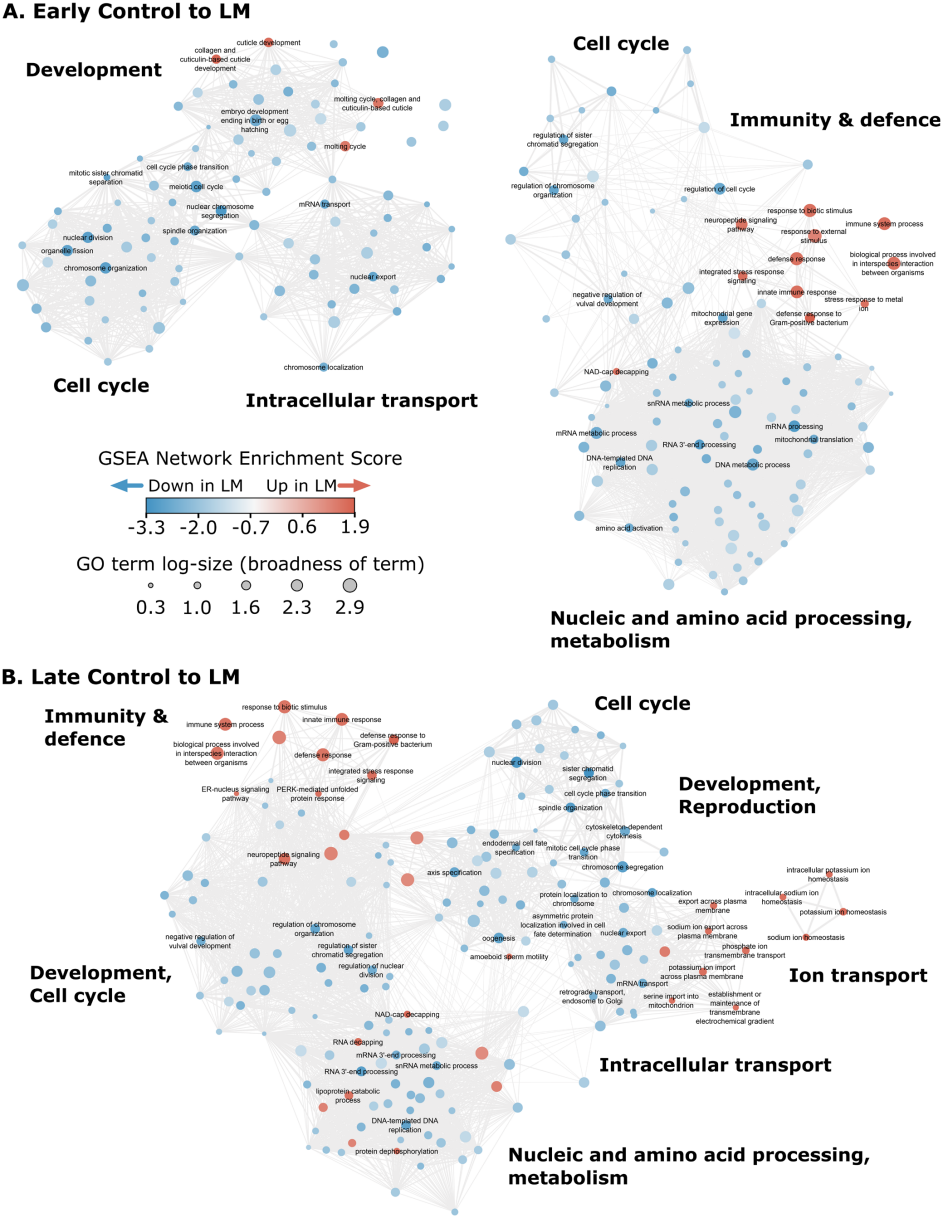
Virulent single infection drove major host immunity‐reproduction fitness trade‐offs. Enriched biological process GO terms between LM (i.e., *L. musarum* alone) and control treatments detected by GSEAs during early (A) and late (B) infection. At both time points, LM infections caused hosts to shift gene expression to increase immunity and defence, while reducing reproduction, development, cell cycle and metabolic processes. GO term nodes are similarity‐filtered by Revigo, showing a representative subsample of all biological process GO terms enriched. Node colour indicates the strength of gene set enrichment, with positive values indicating more highly expressed genes during LM treatment than controls. Node size reflects the broadness of terms by the number of *C. elegans* genes annotated with a given term. Edge width reflects GO term pairwise similarity and is displayed only for the top 3% of edges. The legend applies to both (A) and (B). Sub‐group labels (bold) are summary labels we assigned to categorise perceived functionally related clusters of GO terms. For clarity, only the top 25 (by enrichment score) GO terms for upregulated and downregulated genes are displayed.

The strongest and most consistent transcriptional changes in hosts exposed to the *L. musarum* parasite indicated upregulation of immune and stress response genes coupled with downregulation of reproductive investment genes. Overall, *L. musarum* infected hosts (whether LM or coinfection) had slightly under 1000 DEGs differentiating them from control nematodes during early infection. By the late timepoint, these DEGs increased to over 2000 (Figure 1C). Many of these DEGs were the same genes, even across timepoints. Among these overlapping DEGs, those with the largest transcriptional changes included genes encoding proteins with immune (e.g., upregulated C50F7.5, *clec‐60*, *spp‐21* and *ugt‐18*) (Irazoqui et al. 2010) and reproductive investment functions (e.g., downregulated *vit‐1*, *vit‐3* and *vit‐4*) (Perez and Lehner 2019), (Table S4). This tension between defence and reproduction was further demonstrated at the gene group level (Figure 2). Indicating strong immune responses, all *L. musarum*‐containing treatments had ‘defense response to Gram‐positive bacterium’ as the most enriched biological process GO term among upregulated genes (Data S2). Simultaneously, reproduction and development GO terms such as ‘oogenesis’ or ‘chromosome segregation’ were enriched among genes more lowly expressed during infections with *L. musarum* (Data S2).

Gene expression modules also highlighted gene groups related to virulent infection, which distinguished LM treatment hosts from control animals. Modules 2, 7, 9 and 10 were all positively correlated to late LM infections (correlation = 0.5, 0.5, 0.5 and 0.6, *p* = 0.002, 0.004, 0.003 and 3e‐4, respectively). We discuss modules 2 and 7 below, as they further contrast LM and coinfection. However, modules 9 and 10 were similarly correlated to late LM or coinfection treatments and covered a range of processes such as stimulus response, neuronal development and carbohydrate metabolism. Module 1 was negatively correlated to late LM treatment, which largely related to metabolic and mitochondrial functions (correlation = −0.6, *p* = 6e‐4).

### 3.3 The Protective Parasite Imposed Sub‐Lethal Defence‐Reproduction Fitness Trade‐Offs

Nematode hosts colonised by protective *L. celer* alone modestly activated defence responses. Early LC infection upregulated 33 DEGs relative to controls, which included transporter/carrier proteins, transferases, cytochromes, a chemosensory receptor, antimicrobial *ilys* lysozymes and infection‐responsive peptides (Gravato‐Nobre et al. 2016; Omi and Pujol 2019). Only a single DEG was downregulated at the early timepoint, a putative collagen gene (Data S4). Late stage LC infection signals for increased host immune and stress responses relative to controls were indicated by upregulated DEGs (*n* = 128) that included those such as *clec*, *spp*, *tsp*, DAF‐16 responsive and CUB domain containing protein genes (Data S4) (Di et al. 2018; Dierking et al. 2016; Ermolaeva and Schumacher 2014; Irazoqui et al. 2010; Martineau et al. 2021; Shivers et al. 2008; Visvikis et al. 2014; Yang et al. 2023). Some immune genes were also downregulated during late LC treatment; downregulated DEGs (*n* = 18) included *pud* genes (responsive to DAF‐2 signalling), a *clec* gene and a gene encoding a limited‐scope antimicrobial lysozyme (Data S4) (Berndt et al. 2024; Ding et al. 2013). Overall, host DEGs induced by *L. celer* were far fewer and less indicative of strong responses than DEGs resulting from LM treatments (Figure 1C and Table S3).

**FIGURE 3:**
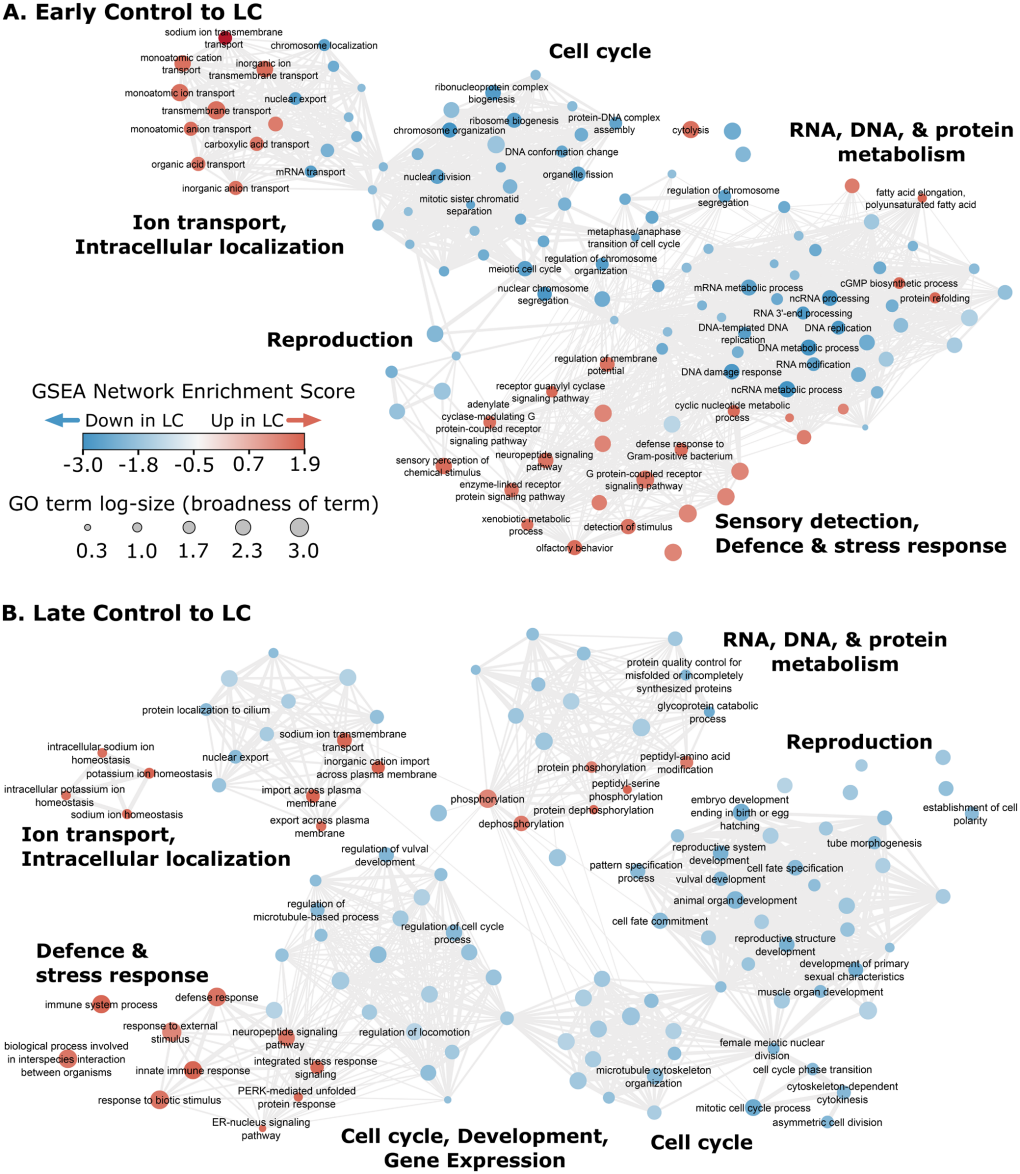
Infection by the protective parasite incurred costs for the host. Enriched biological process GO terms between LC (i.e., *L. celer* alone) and control treatments detected by GSEAs during early (A) and late (B) infection. Both early and late timepoints indicated that *L. celer* infected hosts reduced gene expression related to growth and reproduction compared to time‐matched controls. Simultaneously, infected hosts increased expression of genes related to stress and immune responses. During early infection (A), hosts additionally upregulated genes related to sensory functions, while during late infection (B) signals for reduced reproductive investment were much more numerous. GO term node filtering, figure legend and labelling are as in Figure 2.

More broadly, expression changes at the gene group level indicated LC hosts faced trade‐offs by shifting investment from reproduction and development (downregulated genes) to detection, defence and immunity (upregulated genes) compared to control nematodes. Enriched GO terms selected by a GSEA revealed widespread reduction in gene expression related to host reproduction, cell cycle, growth and development and RNA/protein processing at both early and late LC treatments (Figure 3 and Data S2). During late LC infection, reductions of host reproduction and offspring development were especially apparent. Relative to their time‐matched controls, late LC had ca. 4‐fold more reproduction‐related GO terms enriched among downregulated genes than early LC treatment (Figure 3 and Data S2). LC treatments were also characterised by increased immune and stress response gene expression relative to control nematodes (Figure 3, Data S2). Upregulated processes related to sensory perception and stimulus detection were most clearly associated with early LC treatment rather than late (Figure 3 and Data S2). In total, the degree of change (number of GO terms) was substantially less from controls to LC than controls to either LM or coinfection (Table S2). Similarly, only one WGCNA gene network module clearly differentiated control nematodes and LC hosts, module 2. This module was negatively correlated to LC during early infection, and positively to late LM (Figure 1). Module 2 genes were enriched for GO terms related to RNA processing (Data S2), which although possibly relevant in expressing key genes shaping immunity and fitness, was not highly biologically informative on its own.

### 3.4 The Combination of Coinfection and Endogenous Host Defences Reduced Fitness Trade‐Offs

Host responses to virulent LM infection and attenuated coinfection had largely overlapping gene expression profiles. Only three DEGs were found to distinguish LM and coinfection samples (*far‐3*, *lact‐6* and *spds‐1*, all early infection timepoints and upregulated in coinfection) (Figure 1C) and (Data S4). In contrast, we detected 304 and 1411 DEGs between LC and coinfection treatments at early and late timepoints, respectively (Data S4). This pattern revealed that a largely conserved host transcriptional response to *L. musarum* is activated with or without protection from *L. celer* .

**FIGURE 4:**
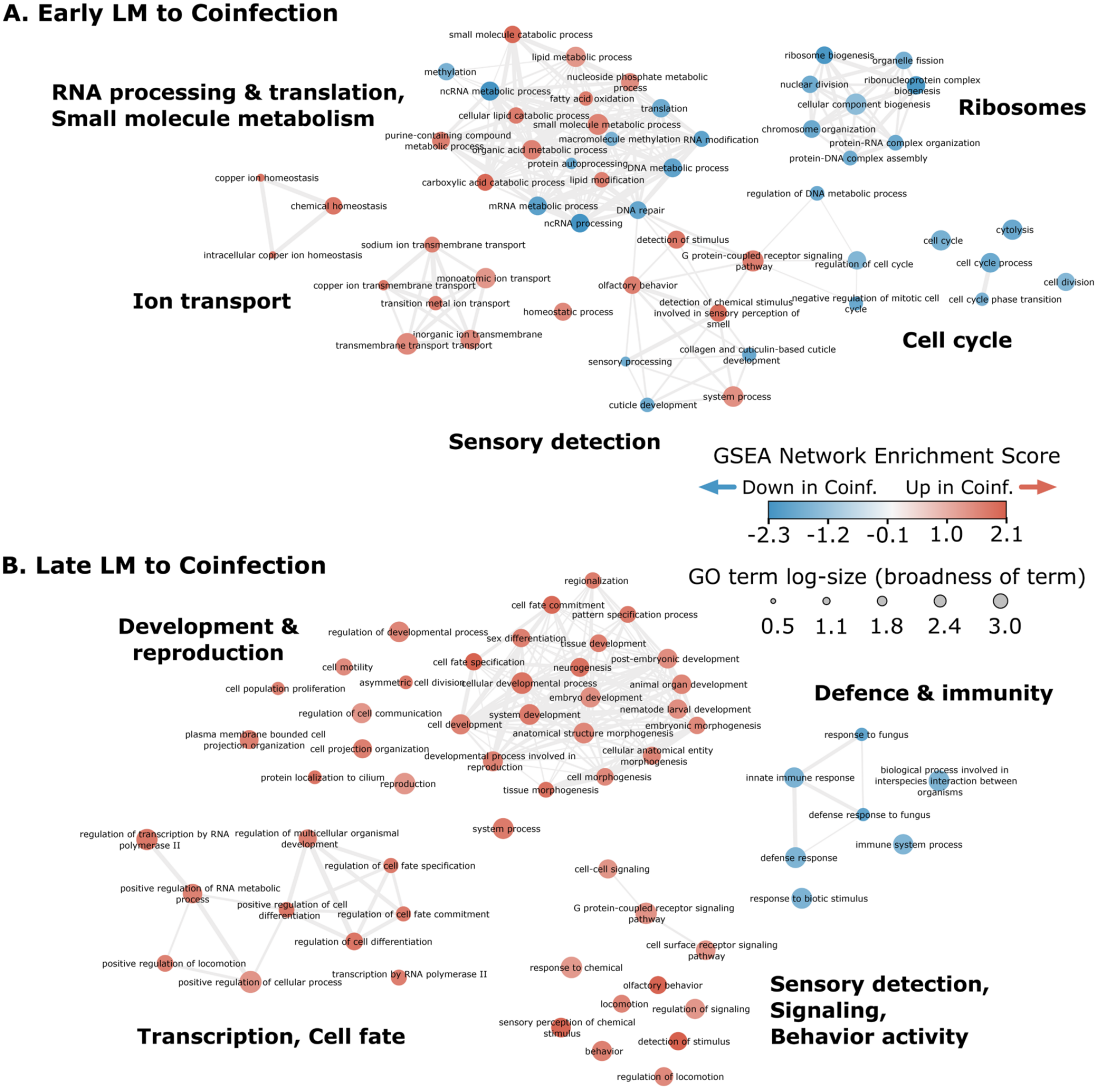
Protective coinfection reduced the severity of host immunity‐reproduction trade‐offs. Enriched biological process GO terms between LM and coinfection treatments detected by GSEAs during early (A) and late (B) infection. Notably, by late infection (B), coinfected hosts appeared to invest less in defence and more in development and reproduction. This shift indicated protective coinfection where *L. celer* prevented severe infection and facilitated greater host investment in direct fitness processes. GO term node filtering, figure legend and labelling are as in Figure 2, with the exception that all nodes are labelled with their GO term.

Although essentially indistinguishable by direct DEG comparisons, changes at the gene group level suggest that LM and coinfected hosts do differ in their defence and reproduction investment. The GSEA and WGCNA approaches detected subtle (below DEG thresholds), but widespread (gene groups), changes in gene transcription between coinfection and LM treatments (Figure 1C). In line with this contrast, DEGs identified between control and LM treatments had similar but marginally less extreme expression values during coinfection (Figure S1). The GSEAs showed coinfected hosts more strongly expressed genes annotated with GO Biological Process terms related to sensory detection than LM‐treated hosts, during early and, especially, late infections (Figure 4). Sensory processes were also identified as responses during early LC, relative to control nematodes (see above, Figure 3) (Data S2). These LC‐associated chemosensory annotations included ‘sensory perception of chemical stimulus’ and ‘olfactory behaviour,’ which were supported by many of the same chemosensory genes activated during coinfection (Figure S2), suggesting a possible role for *L. celer* in promoting protective host stimulus detection.

By late coinfection, hosts appeared to show clear signs of protection with reduced immune gene expression and increased developmental gene expression (compared to nematodes infected by the *L. musarum* parasite alone) (Figure 4). For example, during coinfection, the GO term ‘innate immune response’ was enriched among downregulated genes, which included those encoding putative immune effectors such as lysozymes, C‐type lectin‐like domain proteins, saposins and caenopores and neuropeptide‐like proteins. Simultaneously, these coinfected hosts more highly expressed genes driving enrichments of reproduction GO terms such as ‘embryo development’ and ‘reproduction.’ Many of these genes were annotated for functions such as sperm activation, dermal cell fate, positional development (Hox) and meiosis (Data S2). The differences in late coinfection gene expression connect *L. celer* mediated protection and host capacity to allocate more resources from immune responses to continued development and reproduction.

Gene network modules also distinguished impacts of the more harmful parasite alone (LM treatment) and lower virulence coinfection. Although LM and coinfection shared correlations to many modules, by the late infection timepoint, four modules differentiated LM and coinfection treatments (modules 2, 3, 6 and 7, totaling 548 genes) (Figure 1). Late LM treatment was positively correlated to activation of immune and stress response functions (module 7) and RNA processing and ribosome metabolism (module 2), as indicated by enriched GO terms (Data S2). Late coinfection was instead positively associated with the detection of chemical stimuli and ATP metabolism (module 6, correlation = 0.7, *p* = 2e‐5). Additionally, late coinfection was negatively correlated to organelle and cellular processes and ribo‐nucleotide complexing (module 3, correlation = −0.5, *p* = 0.001) (Data S2). Of likely relevance to disease biology, the increased activation of defence responses in LM treated hosts (module 7) contrasts with the heightened sensing of the environment by coinfected hosts protected by *L. celer* (module 6). In addition to module‐wide enrichment of GO terms, ‘hub’ genes (ranked by WGCNA module membership scores) can also indicate core functions of a module (Langfelder et al. 2013) and corroborate the enrichment results (Document S1). Notably, late LM and coinfection do overlap in positive correlations to sensory functions through module 10 (coinfection correlation = 0.7, *p* = 5e‐5) and nearly so with defence responses in module 8 (Figure 1B, Data S1 and S2). These different module correlations indicated that coinfection and LM elicited some shared (modules 8 and 10) and some distinct (modules 6 and 7) host responses (Figure 1B).

### 3.5 Bacterial Genomics Suggest Microbial Exploitative Competition May Confer Host Protection

Genome assembly produced one primary circular contig for both *L. celer* (4.1 Mbp) and *L. musarum* (3.4 Mbp), with an additional circular 61 kbp putative plasmid contig in *L. musarum* (Table S5). These new assemblies captured the information contained within existing short‐read *L. celer* and *L. musarum* assemblies (Figure S3) while vastly improving continuity from fragmented 235 and 125 contig assemblies (Clark and Hodgkin 2015), respectively. Genomic annotations offered insights into how these microbes may interact with the host or each other to drive virulence or protection, respectively.

We detected possible proviral and plasmid sequences in *L. musarum*. A 52 kbp (69 genes, 66 protein coding) sequence within the *L. musarum* chromosome is possibly of viral origin (geNomad ‘virus score’ = 0.96). The 61 kbp (62 genes, all protein coding) *L. musarum* contig appears to be a plasmid (‘plasmid score’ = 0.98) (Document S2). The proviral sequence was enriched for the ‘DNA Integration’ GO term (42‐fold enrichment, *p* = 0.0061) relative to the whole *L. musarum* genome (Document S2). Both the putative proviral region and plasmid had approximately half of their genes annotated, much lower than the genome‐wide annotation rate (90%).

We further made a preliminary assessment of possible genes contributing to the establishment of infection and virulence in the two *Leucobacter* genomes. The two *Leucobacter* genomes were very similar in their PathoFact‐predicted ‘virulence factor’ abundance, which includes a broad range of genes possibly related to host–parasite interactions such as cell‐surface elements, transcriptional regulators, secretion machinery and toxins (Table S6) (de Nies et al. 2021). Genes with full support as virulence factors made up approximately 23% of their genomes, with 46% of *Leucobacter* genes having partial support. Of these putative virulence factors, a small number were likely toxins (or toxin‐associated, for example, detoxification pumps), making up slightly over 1% of each of the *Leucobacter* genomes (Data S3). The plasmid and proviral regions of *L. musarum* were not enriched for virulence factors relative to the rest of the genome (Table S6). As a complementary approach, we searched for reciprocal best hits (possible orthologs) between *Leucobacter* protein sequences and the virulence factor database (VFDB). The two *Leucobacter* species shared 169 protein hits in the VFDB, with an additional 88 ( *L. celer* ) and 82 (*L. musarum*) unique to the two species (Table S6 and Data S3). Although, in some cases, very similar sequences that differed by the reference species but shared a gene name contributed to the unique hits (e.g., *E. coli* or *Klebsiella pneumoniae* target sequence for the *fimE* gene). Conservatively selecting only VFDB matched genes that also had full PathoFact support yielded 108 genes in *L. celer* and 113 in *L. musarum*. Using this filtered set of putative *Leucobacter* virulence factor orthologs, we compared the *Leucobacter* species by VFDB functional category annotations (Supplemental Data S3). Of 13 categories we detected, the five categories with the most differences between *L. celer* and *L. musarum* were ‘Effector delivery system,’ ‘Adherence,’ ‘Immune modulation,’ ‘Invasion,’ and ‘Biofilm’ (Table 1, Data S3). Within category ‘Effector delivery system,’ *L. musarum* uniquely matched nine type VII secretion system factors compared to one in *L. celer* (and one shared); this secretion system has many roles, with important implications for virulence (Ates et al. 2016). Among ‘Adherence’ factors, we found genes that may be associated with glycoprotein binding (Foster et al. 2013), with marginally more genes in *L. musarum*. *Leucobacter musarum* also had a larger diversity and number of ‘Immune modulation’ factor subtypes than *L. celer* . However, the clearest differences between species for ‘Immune modulation’ were eight bacterial capsule‐like genes present in *L. celer* that *L. musarum* lacked. ‘Biofilm’ genes were more numerous and diverse in *L. celer* . Both bacteria had low numbers of ‘Invasion’ genes; however, *L. celer* had two more than *L. musarum*, which included virulence factor homologues also associated with bacterial capsules in other species (Fan et al. 2022).

**TABLE 1:** The five VFDB virulence factor categories with the most unique hits contrasting *L. celer* and *L. musarum*.

VFDB functional category	Shared	Unique *L. celer*	Unique *L. musarum*
Effector delivery system	7	10	**18**
Adherence	2	8	**11**
Immune modulation	11	15	**18**
Biofilm	1	**4**	1
Invasion	0	**3**	1

*Note:* Rows are ordered by the difference of unique gene counts, and values in bold indicate which *Leucobacter* species had more annotations for that category.

In addition to putative virulence factors, we identified possible bacterial genes involved in ‘social’ functions that may indicate how these microbes interact and compete (Data S3). We identified 59 putative social genes in *L. celer* and 55 in *L. musarum*. Of these genes, many were annotated with siderophore metabolism, which is linked to microbial cooperation, competition and infection virulence (Khasheii et al. 2021; Kramer et al. 2019; Schalk 2024). We found evidence for siderophore synthesis and transport in both *Leucobacter* genomes. There were appreciable numbers of putative transport genes in both *L. celer* (*n* = 21) and *L. musarum* (*n* = 28). However, in *L. celer* , we annotated 10 genes (across two distinct genomic regions, 16.3 kbp and 10.4 kbp in length) related to siderophore synthesis, with only three such genes in *L. musarum* (within a 19.7 kbp span). In line with a role for metal ions (e.g., iron) in host–parasite virulence and/or parasite–parasite public goods interactions, the majority of siderophore‐related genes had full support from PathoFact for a role in causing virulence.

## 4 Discussion

Coinfecting parasites interact with each other, their host and the environment to drive divergent disease outcomes (Alizon et al. 2013; Balmer et al. 2009; Bell et al. 2006; Bernardo‐Cravo et al. 2020; Ford et al. 2016; King et al. 2016; Lello et al. 2018). When these interactions limit the virulence of an infection, they can take on important beneficial roles in host defence (Alizon 2008; Ashby and King 2017; Hoarau et al. 2020; Shen et al. 2019). A reduction in the damaging impacts of infection and other stressors might reduce the need for hosts to make trade‐offs between defence and other life‐history traits, such as reproduction (Critchlow et al. 2024; Dalldorf et al. 2024; Rodrigues et al. 2021). We simultaneously analysed immune and reproduction gene expression across host transcriptomes to infer fitness trade‐offs with and without protective coinfection. While infection by the protective *L. celer* was slightly harmful, the more virulent *L. musarum* infections drove greater fitness trade‐offs to enhance immune activation and reduce reproduction. Hosts protected by coinfection continued to make similar trade‐offs, but at a reduced level.

Sub‐lethal parasitism by *L. celer* was evidenced by gene expression indicating modest fitness trade‐offs, but less severe than virulent infections by *L. musarum*. These findings support phenotypic observations of this system. Specifically, *L. celer* has been shown to hamper *C. elegans* locomotion, as well as delay generation times by over 25% (Hodgkin et al. 2013). In line with delayed generation times, we observed changes in host transcription linked to reduced reproduction and growth—such as downregulated genes with cell fate commitment and embryo development functions. We further identified host responses such as upregulated chemosensation and stimulus detection, immunity and detoxification genes. Chemosensory detection plays an important role in defence of many taxa (Pélissier et al. 2021; Sarabian et al. 2018), including the *C. elegans* response to bacterial parasites and avoidance behaviour (Anderson and McMullan 2018; Lei et al. 2024; Liu and Sun 2021; Pradel et al. 2007; Schulenburg and Ewbank 2007; Schulenburg and Müller 2004; Zhang et al. 2005). Possibly, avoidance behaviour conferred protection at lower cost than immune activation. Despite *L. celer* appearing to drive host trade‐offs, the scale of these responses were smaller than with lethal *L. musarum* infections. *Leucobacter musarum* infections induced differential expression of 20–30‐fold more host genes than *L. celer* . This trend was echoed at the gene group level as well, with nearly two‐fold more GO terms enriched during virulent infection. Moderate costs from a protector that confers net benefits during coinfection have been observed in other host–parasite interactions as well (Sternberg et al. 2011; Vorburger and Gouskov 2011).

Hosts mounted defensive responses following virulent *L. musarum* infections at a cost to reproduction, with and without coinfection. The host response appeared to contain elements specific to severe disease driven by *L. musarum*. For example, host gene *spp‐21* was strongly upregulated in response to both coinfection and *L. musarum*. Yet, *spp‐21* was not activated by *L. celer* alone, nor are we aware of any clear reports of it playing a role in immune defence against other parasitic bacteria. SPP‐21 belongs to a class of saposin/caenopore pore‐forming antimicrobial proteins, which are often activated in infection and stress‐specific manners (e.g., *spp‐1*, *spp‐3*, *spp‐5* or *spp‐9*) (Alegado and Tan 2008; Hoeckendorf and Leippe 2012; Madhu et al. 2020; Roeder et al. 2010). *Leucobacter musarum* also activated more generalised aspects of host immunity, such as *clec‐60* (C‐type lectin‐like domain protein). This gene encodes an intestinal antimicrobial peptide that is broadly implicated in defence against bacterial parasites (Huang et al. 2020; O'Rourke et al. 2006; Sivamaruthi and Balamurugan 2014). In tandem with increased immune activation, hosts downregulated genes associated with reproductive functions. For example, exposure to *L. musarum* appeared to reduce maternal investment in offspring via low expression of host *vit* vitellogenin yolk protein genes, which has been similarly observed in nematodes fending off other microbial infections (Nhan et al. 2019; Perez and Lehner 2019).

Protective coinfection complemented endogenous host defences and reduced the severity of defence‐reproduction trade‐offs. Infected *C. elegans* hosts modulated the expression of many of the same genes responding to *L. musarum* regardless of coinfection (see above). A similar pattern was observed in a study testing a few selected genes of *C. elegans* during coinfection by different parasites (Essebe et al. 2017). Although when defender microbes were experimentally co‐evolved with a more harmful parasite, protective coinfection did elicit significant changes in host immune gene expression (Ford and King 2021). Here, coinfection significantly altered host gene transcription, but only at the gene group level (thousands of genes tested). We found that the host response was muted with protective *L. celer* coinfection. Mechanistically, this pattern highlights that clear changes in phenotypic outcomes can be linked to diffuse, subtle changes in gene expression rather than major changes in a few well‐known key players (Prado et al. 2021; Reimand et al. 2019; Subramanian et al. 2005). Thus, a single‐gene mechanism may be elusive, but broader processes can reveal the mechanistic basis for phenotypic differences. This effect was especially apparent late in disease, hours before mortality from virulent *L. musarum* infection is typically observed. At that time, coinfected hosts expressed gene groups controlling innate immunity and defence responses less while expressing sensory, developmental and reproductive function genes more. Overall, hosts mount similar defences in response to the more harmful parasite, regardless of protection acting in parallel. The degree of that response and associated fitness trade‐offs, however, are attenuated by protective coinfection.

The concept of trade‐offs between immunity and growth or reproduction is a cornerstone of life history theory (Baucom and De Roode 2011; Huot et al. 2014; Schwenke et al. 2016). Experiments with germline deficient *C. elegans* (Alper et al. 2009; Miyata et al. 2008; Tekippe and Aballay 2010) and other animals (Rae et al. 2012; Rodrigues et al. 2021) have shown reproductively sterile hosts survive at higher rates when exposed to microbial parasites. Furthermore, optimal strategies can depend on many factors such as age, environmental stability, reproductive state or infection severity (Duffield et al. 2017; Foo et al. 2023; Kirk et al. 2021; McHugh et al. 2020; Roach and Smith 2020; Schwenke et al. 2016). Individuals may shift towards defence to protect future reproduction or make ‘terminal investments’ into reproduction at the cost of future survival. *Caenorhabditis elegans* make these terminal investments when subjected to certain types of severe stress (Frazier and Roth 2009; Gulyas and Powell 2022). Alternatively, reduced reproductive investment during initial infection may be compensated for later in life after surviving parasite exposure (Pike et al. 2019). We found that virulent infection upregulated immune responses and downregulated growth and reproduction, indicating a survival rather than terminal investment response. Although protective coinfection drove hosts to express fewer immune genes and more reproduction/growth genes, we do not consider this a case of terminal investment. Rather, hosts appeared to rely on the protector for aspects of their defence, with overall increases in survival (Bates et al. 2021). Such host by protector interactions have also been explored using experimental evolution, where costly fitness trade‐offs selected against host innate immunity in favour of symbiont outsourcing, thereby rerouting host evolutionary trajectories (Bates et al. 2021; Martinez et al. 2016).

Protective coinfection competition and infection virulence may be mediated by shared or distinct mechanisms. As colonisation of the host cuticle is critical for *Leucobacter* infection (Document S3) (Hodgkin et al. 2013; Loer et al. 2015; Muir and Tan 2008; O'Rourke et al. 2023), bacterial adhesion and biofilm functions possibly also indicate a mechanism of competition between *L. celer* and *L. musarum* as they vie for space on host surfaces. Another possible mechanism could be metal ion and siderophore scavenging. *Leucobacter celer* had over three‐fold more siderophore synthesis genes than *L. musarum*. This imbalance could indicate competition between coinfecting *L. celer* and *L. musarum*, whether for uptake of shared siderophores or by blocking metal ions via unshared siderophores (Kramer et al. 2019). In such cases of exploitative competition over public goods (i.e., host surface or siderophores), theory predicts intermediate coinfection virulence between single infections by either the defender or parasite alone (Alizon et al. 2013; Gerardo and Parker 2014), as has been observed with *Leucobacter* coinfection. In contrast, competition to extract vital host resources would predict an overall increase in virulence (Alizon et al. 2013), which we did not observe. Furthermore, unannotated *Leucobacter* genes may produce novel virulence factors that lack clear similarity to known examples. In particular, the plasmid‐like and proviral‐like sequences of the *L. musarum* genome had many unannotated genes and their possible contribution to infection and microbial competition remains unknown.

When hosts face severe fitness trade‐offs between defence and reproduction imposed by parasites, protective coinfections can offer a more favourable balance of costs and benefits (Cesar et al. 2024; King and Bonsall 2017). In some cases, major components of defence can be temporarily outsourced, leading to qualitatively different responses from protected and unprotected hosts (Bates et al. 2021; Ford et al. 2022; Martinez et al. 2016; Rafaluk‐Mohr et al. 2022). Here, we found hosts benefited from protective coinfection. However, they only modestly attenuated their endogenous response rather than outsourcing large aspects of their defence. The parallel actions of conserved host defence and protective coinfection may be possible due to protection relying on direct parasite competition, as opposed to being indirectly host‐mediated. This seemingly reduced role for host–parasite crosstalk during protective coinfection suggests that parasite competition (for public goods) may be a generalizable mode of conferring defence. As coinfections are common, possibly the norm, these types of community‐ecology interactions are likely ongoing and subtly modifying host fitness trade‐offs in nature.

## Author Contributions

K.A.B. and K.C.K. designed the experiments and applied for funding. K.A.B. collected RNA for RNAseq and E.J.S. collected DNA for genome assembly. I.W. conducted the analyses and wrote the first version of the manuscript with revisions by K.A.B. and K.C.K.

## Conflicts of Interest

The authors declare no conflicts of interest.

## Supporting information


**Table S1:** Genome annotation tools used, with brief descriptions.
**Table S2:** Total number of enriched GO terms in pairwise contrasts. Columns treatments 1 and 2 give the contrasting values of one variable (either time or infection).
**Table S3:** Total number of DEGs in pairwise contrasts.
**Table S4:** Overlapping DEGs among the top 25 DEGs relative to controls for all *L. musarum* containing treatments.
**Table S5:** Summary statistics of bacterial genome assemblies.
**Table S6:** Gene counts for putative virulence annotations.
**Figure S1:** Gene expression values relative to time‐matched control treatments for LM and coinfection.
**Figure S2:** The underlying core‐set of genes contributing to GSEA enrichments of chemosensory processes show overlaps and distinct subsets between early LC, early coinfection and late coinfection.
**Figure S3:** Mummer outputs of genome to genome alignments for *L. celer* (A) and *L. musarum* (B).
**Document S1:** WGCNA hub genes.
**Document S2:** GeNomad results.
**Document S3:** *L. celer* infection virulence potential.
**Data S1:** Multi‐tab excel with module‐correlations, and gene‐module membership values.
**Data S2:** Multi‐tab excel, with each GSEA or GO‐enrichment for groups selected by DEGs and WGCNA modules.
**Data S3:** Master annotation file of each bacteria genome (one tab each) and all annotations. An additional tab contains the VFDB type information for top candidate orthologs to genes in the *Leucobacter* genomes.
**Data S4:** DEseq2 output data and our DEG classification with WGCNA module per gene for each relevant pairwise comparison.

## Data Availability

Trimmed RNAseq reads have been uploaded to the NCBI Sequence Read Archive (SRA), under BioProject PRJNA1254775. The *Leucobacter* genome assemblies and read data are available under PRJNA1252759 ( *L. celer* ) and PRJNA1252760 (*L. musarum*).
