# Supplementary material for: Host Transcriptomics Reveal Reduction in Defence‐Reproduction Trade‐Offs During Coinfection
Source: Mol Ecol. 2025 Oct 13;34(21):e70124. doi: 10.1111/mec.70124 (PMC12573734; doi:10.1111/mec.70124)
Supplement: Supplementary file 1 — Table S1: Genome annotation tools used, with brief descriptions. Table S2: Total number of enriched GO terms in pairwise contrasts. Columns treatments 1 and 2 give the contrasting values of one variable (either time or infection). Table S3: Total number of DEGs in pairwise contrasts. Table S4: Overlapping DEGs among the top 25 DEGs relative to controls for all L. musarum containing treatments. Table S5: Summary statistics of bacterial genome assemblies. Table S6: Gene counts for putative virulence annotations. Figure S1: Gene expression values relative to time‐matched control treatments for LM and coinfection. Figure S2: The underlying core‐set of genes contributing to GSEA enrichments of chemosensory processes show overlaps and distinct subsets between early LC, early coinfection and late coinfection. Figure S3: Mummer outputs of genome to genome alignments for L. celer (A) and L. musarum (B). Document S1: WGCNA hub genes. Document S2: GeNomad results. Document S3: L. celer infection virulence potential. Data S1: Multi‐tab excel with module‐correlations, and gene‐module membership values. Data S2: Multi‐tab excel, with each GSEA or GO‐enrichment for groups selected by DEGs and WGCNA modules. Data S3: Master annotation file of each bacteria genome (one tab each) and all annotations. An additional tab contains the VFDB type information for top candidate orthologs to genes in the Leucobacter genomes. Data S4: DEseq2 output data and our DEG classification with WGCNA module per gene for each relevant pairwise comparison. [file MEC-34-e70124-s001.docx]

**Supplemental Information:**

**Host transcriptomics reveal reduction in defense-reproduction tradeoffs during coinfection**

Ian Will^1,2*^, Emily J. Stevens^1,3^, Kayla C. King^1,2,4,$^ and Kieran A. Bates^1,5$^*

^1^ Department of Biology, University of Oxford, Oxford, United Kingdom

^2^ Department of Zoology, University of British Columbia, Vancouver, Canada

^3^ School of Life Sciences, Keele University, Newcastle-under-Lyme, United Kingdom

^4^ Department of Microbiology & Immunology, University of British Columbia, Vancouver, Canada

^5^ Blizard Institute, Faculty of Medicine and Dentistry, Queen Mary University of London, London, United Kingdom

^$^ equal contribution

* corresponding authors: IW ian.will@ubc.ca, KAB k.bates@qmul.ac.uk

ORCiD: IW 0000-0002-2396-6778, EJS 0000-0002-5299-4401, KCK 0000-0003-1393-9220, KAB 0000-0003-1559-6014

**Table S1. Genome annotation tools used, with brief descriptions.**

| Tool | Function | Notes |
| --- | --- | --- |
| PGAP | Structural annotation (protein and RNA genes), gene description, taxonomic validation | These annotations are incorporated into the assembly uploaded to NCBI GenBank |
| BUSCO | Find orthologs of universal genes (BUSCOs) to assess assembly completeness | Used the micrococcales BUSCO database, (gene annotations not retained, only genome-wide score) |
| InterProScan | Protein domains, GO terms | Domain based approach |
| eggNOG-mapper | Gene description and name, GO terms, Protein domains, KEGG pathways | Orthology based approach, used the web-based version |
| geNomad | Proviral and plasmid sequences |  |
| PathoFact | Virulence factors | HMM and random forest analyses to make predictions with more flexibility than the reciprocal best hit approach |
| MMseqs2 | Used to search for reciprocal best hits against the VFDB for virulence factors | MMseqs2 "rbh" function between VFDB "core" dataset and our query proteins, default e-value cutoff is 0.001, likely more conservative than the PathoFact approach |
| SOCfinder | Social genes | KOFAM annotations used, no antismash clusters found |
| FeGenie | Siderophore synthesis genes | Used only for an additional siderophore synthesis gene search, as SOCfinder did not identify any siderophore gene clusters |

**Table S2. Total number of enriched GO terms in pairwise contrasts.** Columns Treatment 1 and Treatment 2 give the contrasting values of one variable (either time or infection). Column Shared variable gives the value of the other, shared variable. Biological Process is abbreviated as “BP” and Molecular Function as “MF”. Terms enriched among genes more highly expressed in Treatment 2 are given as “up” and among genes more lowly expressed as “down”.

| **Treatment 1** | **Treatment 2** | **Shared variable** | **BP up** | **BP down** | **MF up** | **MF down** |
| --- | --- | --- | --- | --- | --- | --- |
| 10 h | 20 h | control | 262 | 40 | 33 | 22 |
| control | LC | 10h | 73 | 255 | 64 | 53 |
| control | LM | 10h | 25 | 497 | 21 | 85 |
| control | coinfection | 10h | 74 | 687 | 73 | 142 |
| control | LC | 20h | 33 | 227 | 17 | 17 |
| control | LM | 20h | 57 | 416 | 36 | 76 |
| control | coinfection | 20h | 96 | 427 | 60 | 96 |
| LC | LM | 10h | 14 | 106 | 2 | 21 |
| LC | coinfection | 10h | 21 | 324 | 6 | 47 |
| LC | LM | 20h | 49 | 290 | 31 | 51 |
| LC | coinfection | 20h | 138 | 247 | 61 | 46 |
| LM | coinfection | 10h | 61 | 65 | 31 | 14 |
| LM | coinfection | 20h | 97 | 11 | 17 | 4 |

**Table S3. Total number of DEGs in pairwise contrasts.** Columns Treatment 1 and Treatment 2 give the contrasting values of one variable (either time or infection). Column Shared variable gives the value of the other, shared variable. Column DEG regulation follows the order of the treatments such that upregulation indicates increased gene expression in Treatment 2.

| **Treatment 1** | **Treatment 2** | **Shared variable** | **DEG regulation** | **DEG count** |
| --- | --- | --- | --- | --- |
| 10 h | 20 h | control | up | 1,154 |
| 10 h | 20 h | control | down | 1,741 |
| control | LC | 10 h | up | 33 |
| control | LC | 10 h | down | 1 |
| control | LC | 20 h | up | 128 |
| control | LC | 20 h | down | 18 |
| control | LM | 10 h | up | 758 |
| control | LM | 10 h | down | 151 |
| control | LM | 20 h | up | 2,140 |
| control | LM | 20 h | down | 419 |
| LC | LM | 10 h | up | 413 |
| LC | LM | 10 h | down | 275 |
| LC | LM | 20 h | up | 807 |
| LC | LM | 20 h | down | 255 |
| LC | coinfection | 10 h | up | 239 |
| LC | coinfection | 10 h | down | 65 |
| LC | coinfection | 20 h | up | 1,088 |
| LC | coinfection | 20 h | down | 323 |
| LM | coinfection | 10 h | up | 3 |
| LM | coinfection | 10 h | down | 0 |
| LM | coinfection | 20 h | up | 0 |
| LM | coinfection | 20 h | down | 0 |
| control | coinfection | 10 h | up | 823 |
| control | coinfection | 10 h | down | 63 |
| control | coinfection | 20 h | up | 2,009 |
| control | coinfection | 20 h | down | 323 |

**Table S4. Overlapping DEGs among the top 25 DEGs relative to controls for all *L. musarum* containing treatments.** Descriptions are taken from the Alliance of Genome Resources (v. 7.2.0).

| **Gene** | **Description** | **V2 10 h** | **Coinf. 10 h** | **V2 20 h** | **Coinf. 20 h** |
| --- | --- | --- | --- | --- | --- |
| C50F7.5 | Basic proline-rich protein | Up | Up | Up | Up |
| clec-60 | C-type lectin 60 | Up | Up | Up | Up |
| nspg-7.2 | Nematode Specific Peptide family 7.2 | Up | Up | Up | Up |
| spp-21 | Saposin-like Protein family 21 | Up | Up | Up | Up |
| T27C5.8 | Claudin-like in Caenorhabditis 21 (clc-21) | Up | Up | Up | Up |
| ugt-18 | UDP-glucuronosyltransferase 18 | Up | Up | Up | Up |
| B0563.9 | C-type lectin domain-containing protein | Up | Up | Not top 25 | Not top 25 |
| clec-174 | C-type lectin 174 | Up | Up | Not top 25 | Not top 25 |
| clx-1 | Collagen sequence X-hybridizing 1 | Up | Up | Not top 25 | Not top 25 |
| ech-9 | Enoyl-coa Hydratase 9 | Up | Up | Not top 25 | Not top 25 |
| F01G10.4 | F01G10.4 | Up | Up | Not top 25 | Not top 25 |
| ifas-1 | Inducible fascin Domain containing 1 | Up | Up | Not top 25 | Not top 25 |
| pals-3 | Protein containing ALS2cr12 (ALS2CR12) signature 3 | Up | Up | Not top 25 | Not top 25 |
| C54F6.18 | Secreted protein | Not top 25 | Not top 25 | Up | Up |
| catp-2 | Cation transporting atpase 2 | Not top 25 | Not top 25 | Up | Up |
| clec-70 | C-type lectin 70 | Not top 25 | Up | Up | Up |
| clec-9 | C-type lectin 9 | Not top 25 | Not top 25 | Up | Up |
| dhs-17 | Dehydrogenases, Short chain 17 | Not top 25 | Not top 25 | Up | Up |
| F09C12.2 | Mitogen-activated protein kinase | Not top 25 | Not top 25 | Up | Up |
| F49H6.13 | Claudin-like in Caenorhabditis 12 (clc-12) | Up | Not top 25 | Up | Up |
| igdb-1 | Immunoglobulin and DB (Cys-rich) domains 1 | Not top 25 | Not top 25 | Up | Up |
| K01A6.4 | Glycine rich secreted protein | Not top 25 | Not top 25 | Up | Up |
| lys-3 | Lysozyme 3 | Not top 25 | Not top 25 | Up | Up |
| rab-11.2 | RAB family 11.2 | Up | Not top 25 | Up | Up |
| srlf-13 | SXP/RAL-2-Like protein Family 13 | Not top 25 | Not top 25 | Up | Up |
| Y54G2A.57 | CUB_2 domain-containing protein | Not top 25 | Not top 25 | Up | Up |
| Y57E12B.11 | MS related protein | Up | Not top 25 | Up | Up |
| Y60C6A.2 | Venom protein | Up | Not top 25 | Up | Up |
| Y60C6A.3 | Secreted protein | Up | Not top 25 | Up | Up |
| cyp-25A1 | Cytochrome P450 family 25A1 | Down | Down | Down | Down |
| F46F2.3 | Prion-like-(Q/N-rich)-domain-bearing protein | Down | Down | Down | Down |
| vit-3 | Vitellogenin structural genes (yolk protein genes) 3 | Down | Down | Down | Down |
| vit-4 | Vitellogenin structural genes (yolk protein genes) 4 | Down | Down | Down | Down |
| Y48E1B.8 | Nucleotide-diphospho-sugar transferase domain-containing protein | Down | Down | Down | Down |
| C50E3.12 | C50E3.12 | Down | Down | Not top 25 | Not top 25 |
| F56A4.3 | GST N-terminal domain-containing protein | Down | Down | Not top 25 | Not top 25 |
| hacd-1 | Hydroxy-Acyl-coa Dehydrogenase 1 | Down | Down | Not top 25 | Not top 25 |
| K09C4.5 | Major facilitator superfamily (MFS) profile domain-containing protein | Down | Down | Not top 25 | Not top 25 |
| acp-6 | Acid Phosphatase family 6 | Not top 25 | Not top 25 | Down | Down |
| col-101 | Collagen 101 | Not top 25 | Not top 25 | Down | Down |
| col-43 | Collagen 43 | Not top 25 | Not top 25 | Down | Down |
| D1086.3 | DUF19 domain-containing protein | Not top 25 | Not top 25 | Down | Down |
| F56D6.9 | Glycine-rich cell wall structural protein | Down | Not top 25 | Down | Down |
| grd-4 | Groundhog (hedgehog-like family) 4 | Not top 25 | Not top 25 | Down | Down |
| nhr-235 | Nuclear Hormone Receptor family 235 | Not top 25 | Not top 25 | Down | Down |
| nhr-74 | Nuclear Hormone Receptor family 74 | Not top 25 | Not top 25 | Down | Down |
| pud-3 | Protein Up-regulated in Daf-2(gf) 3 | Not top 25 | Not top 25 | Down | Down |
| ugt-32 | UDP-glucuronosyltransferase 32 | Down | Not top 25 | Down | Down |
| vit-1 | Vitellogenin structural genes (yolk protein genes) 1 | Down | Not top 25 | Down | Down |
| Y51H7C.1 | CLIP domain-containing serine protease | Not top 25 | Not top 25 | Down | Down |
| Y53G8AL.3 | CUB domain-containing protein | Not top 25 | Not top 25 | Down | Down |

**Table S5.** **Summary statistics of bacterial genome assemblies.** Both genomes are highly complete and are composed of either a single (*L. celer*) or two (*L. musarum*) circular contigs. The Actinomycetota are characteristically high GC content bacteria, and these assemblies are within the typical GC content range (Gao & Gupta 2012). In total, *L. celer* had 3,604 and *L. musarum* had 3,131 total genes (including non-protein coding genes, e.g., tRNA genes). We considered a gene annotated if it had any of: gene names/descriptions, PFAM domains, or GO terms (using PGAP, eggNOGmapper, and InterProScan tools). The average nucleotide identity (ANI) calculated by PGAP is relative to the previous RefSeq genome for each species (*L. celer* GCF_001273835.1 and *L. musarum* GCF_001273845.1) and shows strong sequence agreement between assembly methods. Indicating highly complete assemblies, column “BUSCO% s, d, f” reports the proportion of complete-single, complete-duplicated, and fragmented BUSCOs, respectively. We have identified *L. musarum* contig2 as a putative plasmid using geNomad and report its size, GC content, and sequencing depth separately from the *L. musarum* chromosome (contig1).

| **species** | **size (Mbp)** | **GC%** | **average depth** | **protein genes (annotated)** | **ANI** | **BUSCO% s, d, f** |
| --- | --- | --- | --- | --- | --- | --- |
| *L. celer* | 4.1 | 69.2 | 119x | 3,523 (270, 92.3%) | 99.99 | 97.8, 0.6, 0.4 |
| *L. musarum* | 3.4 (contig1),  0.06 (contig2) | 66.9 (contig1),  63.1 (contig2) | 109x (contig1),  127x (contig2) | 3,062 (316, 90.0%) | 100.00 | 98.0, 0.6, 0.4 |

**Table S6. Gene counts for putative virulence annotations.** Virulence factor (“Vir. factor”) and toxin annotations from PathoFact can either have “full” support (both protein model and random forest support) or “part” support from only one sub-analysis. The subset of likely secreted or membrane-associated proteins are given in parentheses “(sec.)”. Column “VFDB RBH” refers to a reciprocal best hit search against the VFDB, yielding possible orthologs to known virulence factors. Column values are gene counts, with a percentage relative to the number of total protein coding genes in that sequence. Sequence “*L. musarum*, primary” refers to the chromosomal sequence excluding the proviral integration.

| **sequence** | **vir. factor, full (sec.)** | **vir. factor, part (sec.)** | **toxin, full (sec.)** | **toxin, part (sec.)** | **VFDB RBH** |
| --- | --- | --- | --- | --- | --- |
| *L. celer* genome | 810, 22.9% (180) | 1,652, 46.9% (188) | 19, 0.5% (5) | 28, 0.8% (3) | 261, 7.4% |
| *L. musarum*, primary | 673, 22.9% (145) | 1,356, 46.2% (136) | 21, 0.7% (6) | 17, 0.6% (2) | 252, 8.6% |
| *L. musarum*, proviral | 2, 3.0% (0) | 33, 50.0% (5) | 0 | 0 | 0 |
| *L. musarum*, plasmid | 11, 17.7% (3) | 9, 15.5% (0) | 0 | 0 | 2, 3.2% |


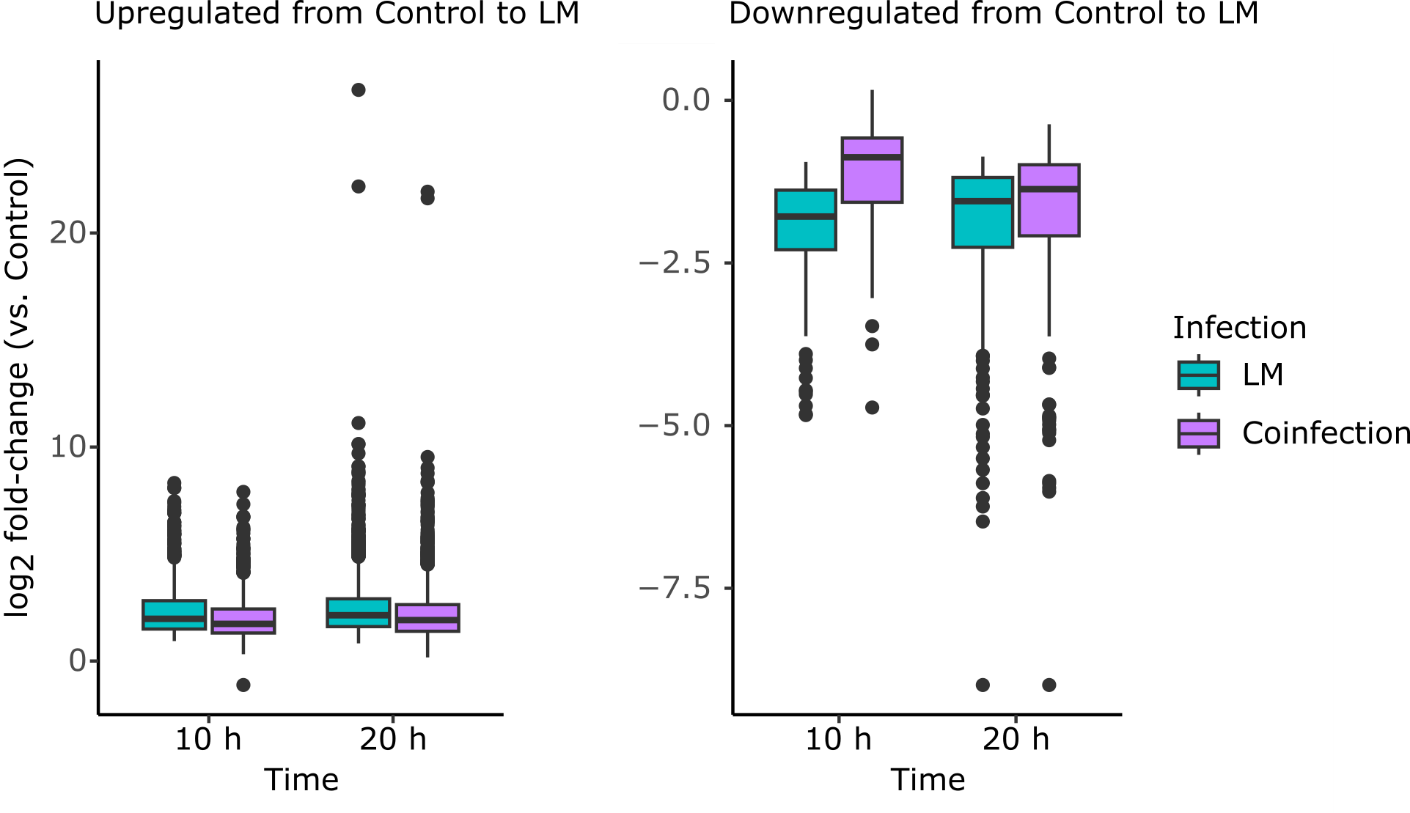


**Figure S1. Gene expression values relative to time-matched control treatments for LM and coinfection.** Genes were selected by control to LM DEG classification. Within time and class (up- or down-regulated), we found control to coinfection expression values to be largely similar to LM. However, importantly, we also observed a minor trend of coinfection expression to be less extreme (trending closer to zero) than LM. This pattern suggested that coinfection protection does not primarily change the genes involved in the defense response, but rather dampens their level of change. These subtle effects appear to drive the findings of our GSEA analyses during coinfection compared to LM treatment.

**
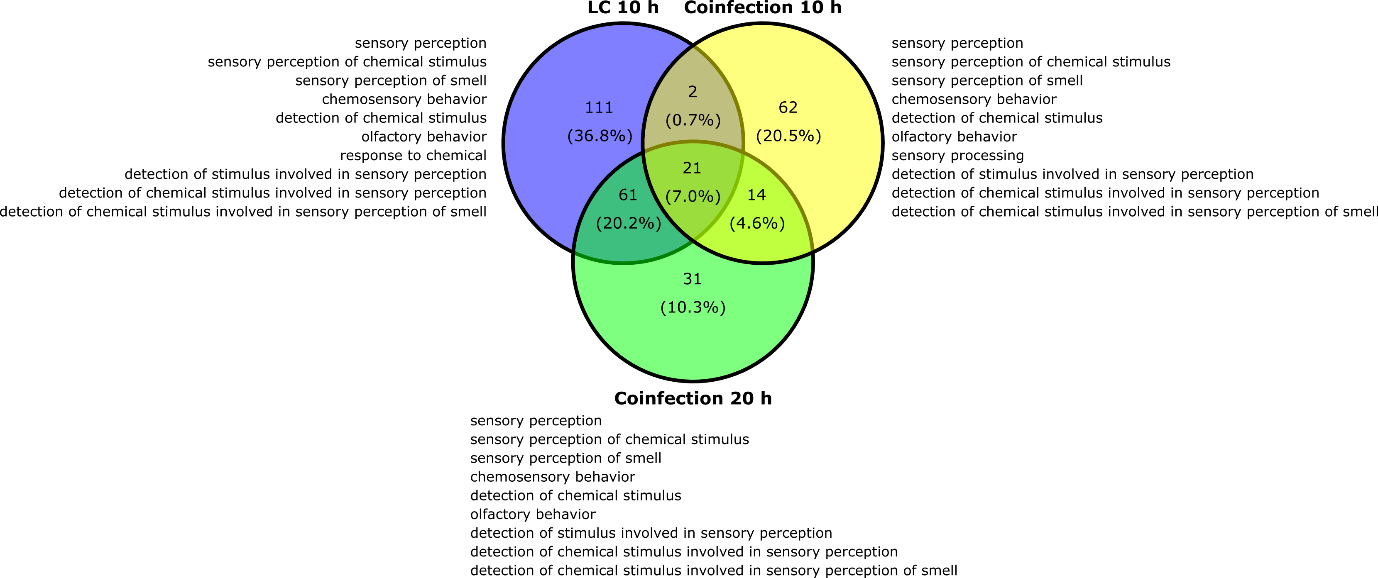
**

**Figure S2. The underlying core-set of genes contributing to GSEA enrichments of chemosensory processes show overlaps and distinct subsets between early LC, early coinfection, and late coinfection.** Core-gene counts are shown in the Venn diagram from GSEAs for LC at 10 h (relative to control at 10 h), coinfection at 10 h (relative to LM at 10 h), and coinfection at 20 h (relative to LM at 20 h). All enriched Biological Process GO terms related to chemosensation are listed by each treatment.

**
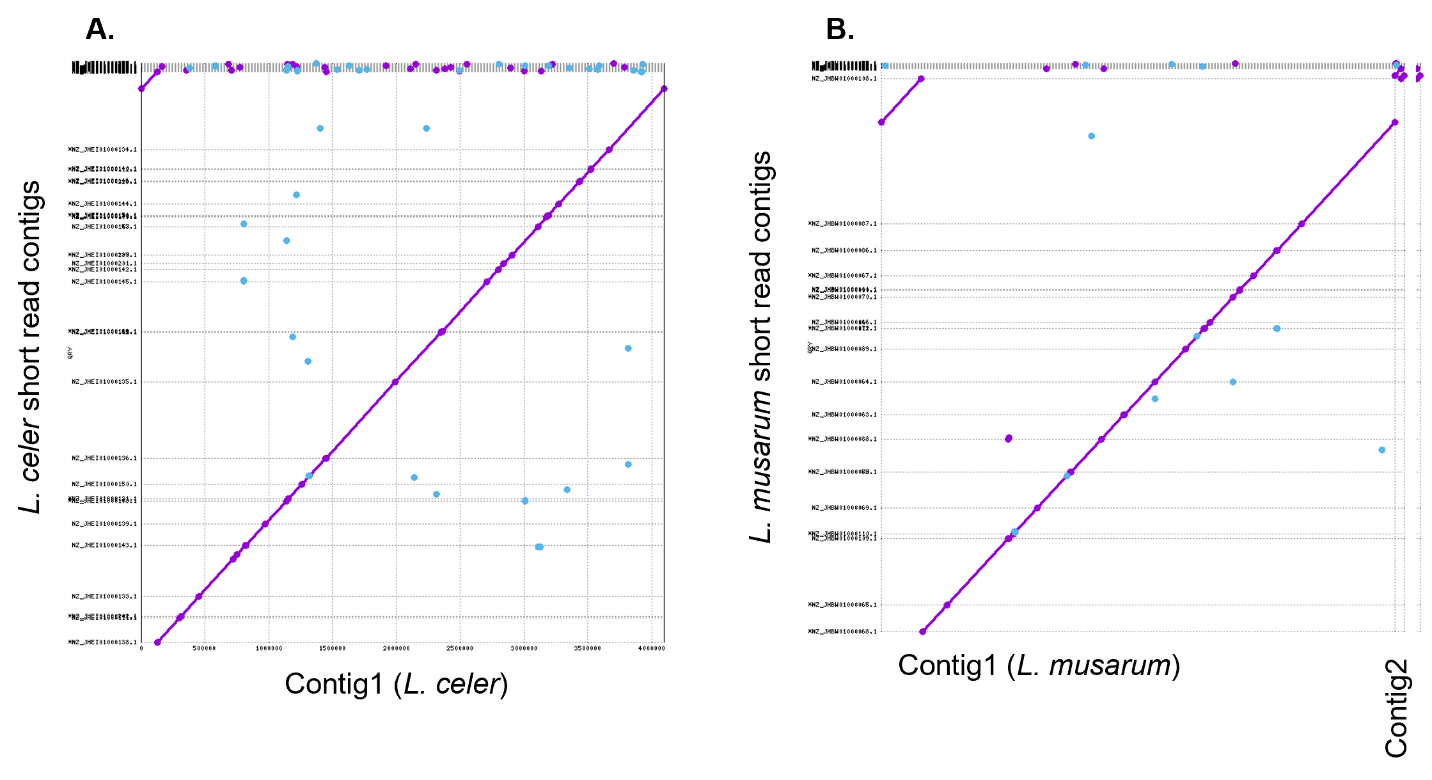
**

**Figure S3.** **Mummer outputs of genome to genome alignments for *L. celer* (A.) and *L. musarum* (B.).** The forward alignment of the short read assembly contigs align well to the continuous long read assemblies (purple) with some short sequences assembled in reverse (blue). Most short read contigs align well, with the smaller poorly resolved fragments scattered across the genomes. One short read contig for each genome can be seen spanning the “start” and “end” of the closed circular assemblies (topmost short read contig line for each plot). Plots were produced with MUMmer (v. 4.0.0rc1) (Marçais *et al.* 2018).

**Supplemental Document S1.**

Among the top 10 hub genes in module 7, we found multiple genes that have been correlated to stress and immune response processes. For example, these hub genes encode the putative amine oxidase C34H4.1 (Otarigho *et al.* 2024), CUB-like domain containing protein C34H4.2 (Pellegrino *et al.* 2014), intestinally expressed Y47H10A.5 (Brunquell *et al.* 2016; Corrêa *et al.* 2010), and a pathogen-signal responsive EB domain protein F10E9.12 (Pukkila-Worley *et al.* 2011) (Supplemental Data S1). Similarly, the top 10 hub genes of module 6 reflected the enrichment of sensory processes. Highlighting stimulus detection we found receptor proteins (e.g., G-protein coupled receptors) *str-13*, *srh-99*, and F09C6.16 (Van Ham *et al.* 2008; Robertson 2000), and C13A2.1, which appears to act downstream of the bacterial-xenobiotic responsive transcription factor AHR-1 (Larigot *et al.* 2022).

**Supplemental Document S2.**

The putative provirus region in *L. musarum* was classified with a “virus score” of 0.96 (scale 0 – 1) and spanned 52 kbp with 69 genes (66 protein coding), 34 of which were viral marker genes, and seven of those 32 were strong “hallmarks” of viral sequences, e.g., “Phage major capsid protein E.” The origin of this proviral sequence may be a type of Caudoviricetes. Indicating proviral genome integration mechanisms, we found the putative proviral region to be enriched for the “DNA integration” Biological Process term relative to the entire *L. musarum* genome (42-fold enrichment, p = 0.0061, annotated to two genes in the provirus sequence using InterPro domain-based GO terms). No other Biological Process enrichments were found. The products of many of these 66 protein genes remain unknown with 48% lacking gene descriptions, GO terms, and PFAM domains – markedly higher than the whole genome with only 10% unannotated genes.

The 61 kbp *L. musarum* contig classified as a plasmid (“plasmid score” = 0.98) contained 62 genes (all protein coding), 19 of which were plasmid markers (with one “hallmark,” matching the type VII secretion *eccE* gene). Most genes (63%) did not have informative annotations, which again was higher than the genome-wide unannotated rate (10%). We did not find any enriched Biological Process GO terms relative to the whole *L. musarum* genome. The presence of putative type II toxin-antitoxin genes (Supplemental Data S3) in this sequence further suggests this contig represents a plasmid and that a toxin-antitoxin mechanism may contribute to plasmid maintenance in *L. musarum* (Zhang *et al.* 2020).

**Supplemental Document S3.**

Although *L. celer* acts as a defensive symbiont in the context we studied here, this protector acts as a parasite with different host genotypes, in other environments, and in the absence of *L. musarum* (Bates *et al.* 2021; Hodgkin *et al.* 2013; O’Rourke *et al.* 2023). The *Leucobacter* species differ in their capacity to attach to hosts. Mutant *C. elegans* with altered cuticle surface glycosylation (e.g., *srf* or *bus* gene mutants) can have radically altered susceptibilities to *Leucobacter* infection – with tradeoffs leading to host death with *L. celer* infection and survival of *L. musarum* exposure (Bates *et al.* 2021; Hodgkin *et al.* 2013; Loer *et al.* 2015; O’Rourke *et al.* 2023). In line with a key role for host surfaces mediating infection, the *Leucobacter* species differed by putative adhesion genes (e.g., lectin-like genes) and bacterial capsule-like genes, which could relate to cell-surface functions such as adhesion, biofilm, and immune evasion/modulation (Gao *et al.* 2024). The activity of such adhesion genes in *L. celer* is one possible mechanism to explain reduced *C. elegans* locomotion on bacterial lawns and the binding together of hosts in lethal “worm stars” during liquid culture (Hodgkin *et al.* 2013). Adhesion also appears important for other *Leucobacter* parasites. *Leucobacter chromiireducens* subsp. *solipictus*, drives lethal uterine infections in *C. elegans* and biofilms possibly play a role (Muir & Tan 2008).

**Supplemental Information References**

Bates, K.A., Bolton, J.S. & King, K.C. (2021). A globally ubiquitous symbiont can drive experimental host evolution. *Mol Ecol*, 30, 3882–3892.

Brunquell, J., Morris, S., Lu, Y., Cheng, F. & Westerheide, S.D. (2016). The genome-wide role of HSF-1 in the regulation of gene expression in Caenorhabditis elegans. *BMC Genomics*, 17, 1–18.

Corrêa, R.L., Steiner, F.A., Berezikov, E. & Ketting, R.F. (2010). MicroRNA–Directed siRNA Biogenesis in Caenorhabditis elegans. *PLoS Genet*, 6, 1000903.

Gao, B. & Gupta, R.S. (2012). Phylogenetic Framework and Molecular Signatures for the Main Clades of the Phylum Actinobacteria. *Microbiol Mol Biol Rev*, 76, 66.

Gao, S., Jin, W., Quan, Y., Li, Y., Shen, Y., Yuan, S., *et al.* (2024). Bacterial capsules: Occurrence, mechanism, and function. *npj Biofilms and Microbiomes 2024 10:1*, 10, 1–15.

Van Ham, T.J., Thijssen, K.L., Breitling, R., Hofstra, R.M.W., Plasterk, R.H.A. & Nollen, E.A.A. (2008). C. elegans Model Identifies Genetic Modifiers of α-Synuclein Inclusion Formation During Aging. *PLoS Genet*, 4, 1000027.

Hodgkin, J., Félix, M.A., Clark, L.C., Stroud, D. & Gravato-Nobre, M.J. (2013). Two Leucobacter Strains Exert Complementary Virulence on Caenorhabditis Including Death by Worm-Star Formation. *Current Biology*, 23, 2157–2161.

Larigot, L., Bui, L.C., de Bouvier, M., Pierre, O., Pinon, G., Fiocca, J., *et al.* (2022). Identification of Modulators of the C. elegans Aryl Hydrocarbon Receptor and Characterization of Transcriptomic and Metabolic AhR-1 Profiles. *Antioxidants*, 11, 1030.

Loer, C.M., Calvo, A.C., Watschinger, K., Werner-Felmayer, G., O’Rourke, D., Stroud, D., *et al.* (2015). Cuticle integrity and biogenic amine synthesis in Caenorhabditis elegans require the cofactor tetrahydrobiopterin (BH4). *Genetics*, 200, 237–253.

Marçais, G., Delcher, A.L., Phillippy, A.M., Coston, R., Salzberg, S.L. & Zimin, A. (2018). MUMmer4: A fast and versatile genome alignment system. *PLoS Comput Biol*, 14, e1005944.

Muir, R.E. & Tan, M.W. (2008). Virulence of Leucobacter chromiireducens subsp. solipictus to Caenorhabditis elegans: Characterization of a novel host-pathogen interaction. *Appl Environ Microbiol*, 74, 4185–4198.

O’Rourke, D., Gravato-Nobre, M.J., Stroud, D., Pritchett, E., Barker, E., Price, R.L., *et al.* (2023). Isolation and molecular identification of nematode surface mutants with resistance to bacterial pathogens. *G3: Genes|Genomes|Genetics*, 13, 56.

Otarigho, B., Butts, A.F. & Aballay, A. (2024). Neuronal NPR-15 modulates molecular and behavioral immune responses via the amphid sensory neuron-intestinal axis in C. elegans. *bioRxiv*.

Pellegrino, M.W., Nargund, A.M., Kirienko, N. V., Gillis, R., Fiorese, C.J. & Haynes, C.M. (2014). Mitochondrial UPR-regulated innate immunity provides resistance to pathogen infection. *Nature*, 516, 414.

Pukkila-Worley, R., Ausubel, F.M. & Mylonakis, E. (2011). Candida albicans Infection of Caenorhabditis elegans Induces Antifungal Immune Defenses. *PLoS Pathog*, 7, e1002074.

Robertson, H.M. (2000). The Large srh Family of Chemoreceptor Genes in Caenorhabditis Nematodes Reveals Processes of Genome Evolution Involving Large Duplications and Deletions and Intron Gains and Losses. *Genome Res*, 10, 192–203.

Zhang, S.-P., Wang, Q., Quan, S.-W., Yu, X.-Q., Wang, Y., Guo, D.-D., *et al.* (2020). Type II toxin–antitoxin system in bacteria: activation, function, and mode of action. *Biophysics Reports 2020 6:2*, 6, 68–79.
